# Annual acknowledgement of manuscript reviewers

**DOI:** 10.1186/1471-2415-13-7

**Published:** 2013-04-05

**Authors:** Emilie Aime

**Affiliations:** 1 BioMed Central, Floor 6, 236 Gray’s Inn Road, London WC1X 8HB, UK

## Abstract

**Contributing reviewers:**

The editors of *BMC Ophthalmology* would like to thank all of our reviewers who have contributed to the journal in volume 12 (2012).

## 

Seyed-Hossein Abtahi

Iran

Khaled Abu-Amero

Saudi Arabia

Girdhar Agarwal

India

Amar Agarwal

India

Anita Agarwal

USA

Tan Aik Kah

Malaysia

Jose Leopoldo Ferreira Antunes

Brazil

Paul Appleby

UK

James Aquavella

USA

Neil Archibald

UK

Navid Ardjomand

Austria

Francisco Ascaso

Spain

Jatin Ashar

India

Huban Atilla

Turkey

Sonia Attia

Tunisia

Teresio Avitabile

Italy

Augusto Azuara-Blanco

UK

Francesco Bandello

Italy

Ante Barisić

Croatia

Keith Barton

UK

Sherry Bass

USA

David Beebe

USA

Arthur Bergen

Netherlands

Ke Bilian

China

Luigi Bisceglia

Italy

Justin Blackburn

USA

Puran Bora

USA

Rupert Bourne

UK

Christopher Bowd

USA

Fredric Brandt

USA

Peter Calabresi

USA

Ricardo Casaroli

Spain

Robin Casten

USA

Santam Chakraborty

Canada

David Chang

USA

Balwantray Chauhan

Canada

Bo Young Chun

South Korea

Sung Kun Chung

South Korea

Salvatore Cillino

Italy

Srdjan Cirovic

UK

Grant Comer

USA

Paul Courtright

Tanzania

David Crabb

UK

Alan Crandall

USA

Annegret Dahlmann-Noor

UK

Ramin Daneshvar

Iran

Leon Davies

UK

Giuseppe De Crecchio

Italy

Margaret Deangelis

USA

Iva Dekaris

Croatia

Alastair Denniston

UK

Efstathios Detorakis

Greece

Anuradha Dhingra

USA

Serge Doan

France

James Dunn

USA

Charles Eberhart

USA

David Elliott

UK

Sarah Ennis

UK

Casper Erkeelns

Netherlands

Julio Escribano

Spain

Jennifer Evans

UK

Antonio Ferreras

Spain

Robert Finger

Australia

Christina Flaxel

USA

Raimondo Forte

Italy

Akbar Fotouhi

Iran

Ellen Freeman

Canada

Rudolf Fuchshofer

Germany

Nobuo Fuse

Japan

John Galaznik

USA

Paul Gamlin

USA

Jose Javier Garcia-Medina

Spain

Jian Ge

China

Gerasimos Georgopoulos

Greece

Alireza Ghaffarieh

USA

Sambuddha Ghosh

India

Parikshit Gogate

India

David Goldblum

Switzerland

J Gonzalez-Meijome

Portugal

Bamini Gopinath

Australia

Michalina Gora

USA

Irene Gottlob

UK

Franz Grehn

Germany

Matthias Christian Grieshaber

Switzerland

Yan Guex-Crosier

Switzerland

Freedom Nkhululeko Gumedze

South Africa

Pinakin Gunvant

USA

Bhaskar Gupta

UK

Abigail Hackam

USA

Kalish Hadas

Israel

Mohammad Haider

Kuwait

Gabor Michael Halmagyi

Australia

Chris Hammond

UK

Marcelo Hatanaka

Brazil

Sohan Hayreh

USA

John Heckenlively

USA

Steffen Heegaard

Denmark

Javad Heravian

Iran

Sergio Hernandez-Da Mota

Mexico

Taichi Hikichi

Japan

Kazuyuki Hirooka

Japan

Yhu-Chering Huang

Taiwan

Jean-Pierre Hubschman

USA

Jacques Hugon

France

Young Hoon Hwang

South Korea

Pierluigi Iacono

Italy

Parul Ichhpujani

India

Michele Iester

Italy

Milko Iliev

Switzerland

Ugo Introini

Italy

Rafael Iribarren

Argentina

Tatsuro Ishibashi

Japan

Amirul Islam

Australia

Muhammad Ismail

Pakistan

Christina Jacobi

Germany

Yaping Jin

Canada

Sven Jonuscheit

UK

Jorge Jorge

Portugal

Hayyam Kıratlı

Turkey

Ramazan Kahveci

Turkey

Dimitrios Karagiannis

Greece

Niral Karia

UK

Niro Kasahara

Brazil

Satoru Kase

Japan

Seema Kashyap

India

Inderjeet Kaur

India

Tatsushi Kawaguchi

Japan

Tetsuya Kawakita

Japan

Jill Keeffe

Australia

Simon Kelly

UK

Ahmad Kheirkhah

Iran

Daniel Kiernan

USA

Jens Folke Kiilgaard

Denmark

Chan Yun Kim

South Korea

Jonathan King

USA

George Kitsos

Greece

Mark Kleinman

USA

Hideki Koizumi

Japan

Haris Kokotas

Greece

Aart Kooijman

Netherlands

Koung Hoon Kook

South Korea

Vassilios Kozobolis

Greece

Hatem Krema

Canada

Kazuyuki Kumagai

Japan

Sentaro Kusuhara

Japan

Georgios Labiris

Greece

Timothy Lai

Hong Kong

Jimmy Lai

Hong Kong

Augustinus Laude

Singapore

Michael Lemp

USA

Dinko Leovic

Croatia

Dexter Yu Lung Leung

Hong Kong

Jaime Levy

Israel

Yuanbo Liang

China

Shu Lang Liao

Taiwan

Sue Lightman

UK

Longqian Liu

China

Nicola Logan

UK

Antonio Longo

Italy

Louise Longworth

UK

Da-Wen Lu

Taiwan

Peirong Lu

China

Ted Maddess

Australia

Thomas Mader

USA

Matthias Maier

Germany

Raffaele Mancino

Italy

Xiao Wen Mao

USA

Michael Marmor

USA

Lene Martin

Sweden

Raul Martin

Spain

Suzana Matayoshi

Brazil

Scott Mcclatchey

USA

Charles Mcmonnies

Austria

Pauline Mcnamee

UK

Sounira Mehri

Tunisia

Bouzid Menaa

Belgium

Rita Mencucci

Italy

Javier Mendicute

Spain

Roberto Mendoza-Londono

Canada

Catherine Meyerle

USA

Jonathan Micieli

Canada

Tatsuya Mimura

USA

Sasan Moghimi

Iran

Mehrdad Mohammadpour

Iran

Elad Moisseiev

Israel

Javier Montero

Spain

Ramana Moorthy

USA

Naoyuki Morishige

Japan

Caleb Mpyet

Nigeria

Arijit Mukhopadhyay

India

Ian Murdoch

UK

Kei Nakai

Japan

Mitsuru Nakazawa

Japan

Vinay Nangia

India

Giorgio Napolitano

Italy

Raja Narayanan

India

Gabor Nemeth

Hungary

Dan Nguyen

UK

Shufang Nie

USA

Paolo Nucci

Italy

Kyoko Ohno-Matsui

Japan

Kouichi Ohta

Japan

Michael O’Keeffe

Ireland

Yoko Okunuki

Japan

Sotaro Ooto

Japan

Neville Osborne

UK

Luis Pablo

Spain

Mohammad Pakravan

Iran

Ki Ho Park

South Korea

Kyu Hyung Park

South Korea

Avinash Pathengay

India

Carlos Pavesio

UK

Antonio Pinna

Italy

Augusto Pocobelli

Italy

Dimitra Portaliou

Greece

Pawan Prasher

India

Paul-Rolf Preußner

Germany

Marianne Price

USA

Caroline Quach

Canada

Rony Rachmiel

Israel

Mohammad Taher Rajabi

Iran

Zhale Rajavi

Iran

Jagat Ram

India

Guglielmo Ramieri

Italy

Pradeep Ramulu

USA

Aparna Rao

India

Ravindran Ravilla

India

Mohammad Reza Razeghinejad

Iran

Sagili Chandrasekhara Reddy

Malaysia

Ashok Reddy

India

Richard Redmond

UK

Caio Regatieri

Brazil

Marek Rekas

Poland

Ramakrishnan Rengappa

India

Rodolfo Repetto

Italy

Thomas Ritter

Ireland

Anthony Robson

UK

Teresa Rolle

Italy

Sylvain Roy

Switzerland

Afsun Sahin

Turkey

Masaaki Saito

Japan

Chameen Samarawickrama

Australia

Krishnaiah Sannapaneni

India

Marcony Santhiago

USA

Hiroaki Satoh

Japan

Giacomo Savini

Italy

Rohit Saxena

India

Leopold Schmetterer

Austria

Mitra Sehi

USA

William Seiple

USA

Masaaki Seki

Japan

Saadettin Sel

Germany

Chirag Shah

USA

Savitri Sharma

India

Justin Sherwin

UK

Wisam Shihadeh

Jordan

Masahiko Shimura

Japan

Takuhei Shoji

Japan

Dhananjay Shukla

India

Tamilvanan Shunmugaperumal

Belgium

Rafael Simo

Spain

Gungor Sobaci

Turkey

Merih Soylu

Turkey

Yasemin Soysal

Turkey

Miles Stanford

UK

David Steel

UK

Michael Stewart

USA

Krishnakumar Subramanian

India

Jaspreet Sukhija

India

Michael Sullivan-Mee

USA

Yasmin Sultana

India

Gangadhara Sundar

Singapore

Kiyoshi Suzuma

Japan

Eszter Szalai

Hungary

Mehryar (Ray) Taban

USA

Colin Tan

Singapore

Jeremiah Tao

USA

Muhittin Taskapili

Turkey

Marie-José Tassignon

Belgium

Sinan Tatlipinar

Turkey

Thomas Theelen

Netherlands

Benjamin Thompson

New Zealand

Peter Bjerre Toft

Denmark

Louis Tong

Singapore

Ole Torffvit

Sweden

Carol Toris

USA

Lisa Toto

Italy

Rong Kung Tsai

Taiwan

Ekaterini Tsilou

USA

Sally Twining

USA

Takashi Ueta

Japan

Usha Kim Usha Kim

India

Ghmb Van Rens

Netherlands

Daniel Vitor Vasconcelos-Santos

Brazil

Federico Velez

USA

Pradeep Venkatesh

India

Johan Verbraecken

Belgium

Manuel Vidal-Sanz

Spain

Andrea L Vincent

New Zealand

A. Wegner

Germany

Shi Weiyun

China

Feng Wen

China

Kirk Wilhelmus

USA

Mark Willcox

Australia

Colin Willoughby

UK

Se Joon Woo

South Korea

Richard Wormald

UK

Kaili Wu

China

Edward Wylegala

Poland

Ozlem Yalcin Tok

Turkey

Lawrence Yannuzzi

USA

Shahin Yazdani

Iran

Chungkwon Yoo

South Korea

Incheon You

South Korea

Hyeong Gon Yu

South Korea

Nurşen Yüksel

Turkey

David Zacks

USA

Hadi Zambarakji

UK

Shamshad Zarina

Pakistan

Andrew Zele

Australia

Qingjiong Zhang

China

Fengju Zhang

China

Yun E Zhao

China

Xiaodong Zheng

Japan

## Pre-publication history

The pre-publication history for this paper can be accessed here:

http://www.biomedcentral.com/1471-2415/13/7/prepub

